# Markers of Systemic Inflammation and Environmental Enteric Dysfunction Are Not Reduced by Zinc or Multivitamins in Tanzanian Infants: A Randomized, Placebo-Controlled Trial

**DOI:** 10.1016/j.jpeds.2019.02.016

**Published:** 2019-07

**Authors:** Jacqueline M. Lauer, Christine M. McDonald, Rodrick Kisenge, Said Aboud, Wafaie W. Fawzi, Enju Liu, Hao Q. Tran, Andrew T. Gewirtz, Karim P. Manji, Christopher P. Duggan

**Affiliations:** 1 Division of Gastroenterology, Hepatology and Nutrition, Boston Children's Hospital, Boston, MA; 2 Children's Hospital Oakland Research Institute, Oakland, CA; 3 Department of Pediatrics and Child Health, Muhimbili University of Health and Allied Sciences, Dar es Salaam, Tanzania; 4 Department of Microbiology and Immunology, Muhimbili University of Health and Allied Sciences, Dar es Salaam, Tanzania; 5 Department of Global Health and Population, Harvard T.H. Chan School of Public Health, Boston, MA; 6 Department of Epidemiology, Harvard T.H. Chan School of Public Health, Boston, MA; 7 Department of Nutrition, Harvard T.H. Chan School of Public Health, Boston, MA; 8 Institutional Centers of Clinical and Translational Research, Boston Children's Hospital, Boston, MA; 9 Institute for Biomedical Sciences, Georgia State University, Atlanta, GA

**Keywords:** anti-flagellin, anti-LPS, alpha-1-acid glycoprotein, C-reactive protein, insulin-like growth factor-1, insulin-like growth factor binding protein-3, AGP, Alpha-1-acid glycoprotein, CRP, C-reactive protein, EED, Environmental enteric dysfunction, IGF-1, Insulin-like growth factor-1, IGFBP-3, Insulin-like growth factor binding protein-3, L:M, Lactulose:mannitol, LMIC, Low- and middle-income country, LPS, Lipopolysaccharide

## Abstract

**Objective:**

To examine whether daily zinc and/or multivitamin supplementation reduce biomarkers of environmental enteric dysfunction (EED), systemic inflammation, or markers of growth in a sample of infants from Dar es Salaam, Tanzania.

**Study design:**

Subgroup analysis of infants participating in a randomized, double-blind, placebo-controlled trial received daily oral supplementation of zinc, multivitamins, zinc + multivitamins, or placebo for 18 months starting at 6 weeks of age. EED (anti-flagellin and anti-lipopolysaccharide immunoglobulins), systemic inflammation (C-reactive protein and alpha-1-acid glycoprotein), and growth biomarkers (insulin-like growth factor-1 and insulin-like growth factor binding protein-3) were measured via enzyme-linked immunosorbent assay in a subsample of 590 infants at 6 weeks and 6 months of age. EED biomarkers also were measured in 162 infants at 12 months of age.

**Results:**

With the exception of anti-lipopolysaccharide IgG concentrations, which were significantly greater in infants who received multivitamins compared with those who did not (1.41 ± 0.61 vs 1.26 ± 0.65, *P* = .006), and insulin-like growth factor binding protein-3 concentrations, which were significantly lower in children who received zinc compared with those who did not (981.13 ± 297.59 vs 1019.10 ± 333.01, *P* = .03), at 6 months of age, we did not observe any significant treatment effects of zinc or multivitamins on EED, systemic inflammation, or growth biomarkers.

**Conclusions:**

Neither zinc nor multivitamin supplementation ameliorated markers of EED or systemic inflammation during infancy. Other interventions should be prioritized for future trials.

**Trial registration:**

Clinicaltrials.gov: NCT00421668.

See editorial, p 8

Children in low- and middle-income countries (LMICs) commonly experience low-grade systemic inflammation, which can suppress the production of insulin-like growth factor-1 (IGF-1) and result in linear growth failure.^1^, ^2^, ^3^ A primary cause of systemic inflammation in young children in LMICs is recurrent acute illness, eg, cough, fever, and diarrheal disease.^4^, ^5^ In addition, systemic inflammation is thought to be both a cause and consequence of environmental enteric dysfunction (EED), a disorder of the small intestine characterized by alterations in the structure and function of the gut, specifically, mucosal inflammation, villous blunting, altered barrier integrity, and reduced absorptive capacity, due to exposure to unhygienic conditions.^6^, ^7^, ^8^ Both EED and systemic inflammation biomarkers have been linked to poor child growth^5^, ^9^, ^10^; however, interventions to successfully ameliorate EED and/or systemic inflammation in settings with chronic malnutrition and exposure to environmental pathogens and toxins remain elusive.

Two potential nutritional interventions for ameliorating EED and/or systemic inflammation include zinc and multivitamin supplementation. Zinc plays an important role in cell-mediated immunity and is an antioxidant and anti-inflammatory nutrient.^11^ Furthermore, zinc may directly affect transepithelial ion transport and promote the maintenance of tight junctions between epithelial cells.^12^ In some animal models, zinc deficiency has been associated with altered immune function^13^, ^14^ as well as both histologic (ie, ulcerations, inflammatory infiltration, and edema of the jejunum)^15^, ^16^ and functional gastrointestinal changes (ie, decreased nutrient absorption).^17^, ^18^

Although the mechanism of action is not completely understood, zinc supplementation has been shown to positively impact linear growth in young children, albeit modestly.^19^, ^20^ Furthermore, zinc supplementation has been shown to reduce the incidence, severity, and duration of diarrheal episodes^21^, ^22^, ^23^, ^24^ and to improve markers of intestinal permeability, particularly in young Bangladeshi children with infectious diarrhea,^25^ as well as in patients with inflammatory lesions such as Crohn's disease.^26^ In a study of young rural Malawian children, therapeutic zinc supplementation (14-day course of 20 mg of zinc sulfate) was shown to attenuate the progression of EED, assessed via the lactulose:mannitol (L:M) ratio, compared with placebo.^27^

There is limited evidence to support the role of multiple vitamins in ameliorating EED or systemic inflammation. Like zinc, numerous water-soluble vitamins have antioxidant and anti-inflammatory properties, and deficiencies have been shown to alter gastrointestinal and systemic immune function.^28^, ^29^ Furthermore, multivitamins were shown to increase villous height and absorptive area in a study of HIV-negative Zambian adults^30^ and to transiently ameliorate EED in a study of young Malawian children.^31^

Although C-reactive protein (CRP) and alpha-1-acid glycoprotein (AGP) are relatively established indicators of systemic inflammation, no single biomarker for EED has been identified and universally accepted. We have previously evaluated anti-flagellin and anti-lipopolysaccharide (LPS) immunoglobulins as potential EED biomarkers. In young Tanzanian children, we have linked greater concentrations of these antibodies to poor child growth,^32^ greater blood pressure in childhood,^33^ as well as alterations in neurodevelopment.^34^ In Ugandan mothers and their infants, we have observed a relationship between these biomarkers and adverse birth outcomes (lower infant length and shorter gestational age at birth).^35^

In our earlier parent clinical trial, we demonstrated that neither daily zinc nor multivitamin supplementation reduced the incidences of underweight, stunting, or wasting in Tanzanian infants.^36^ However, we did observe that zinc supplementation reduced the occurrence of diarrhea and upper respiratory tract infections.^37^ Furthermore, we observed that anti-flagellin and anti-LPS IgA and IgG concentrations were greater in Tanzanian infants compared with healthy controls from Boston and were associated with an increased risk of becoming underweight.^32^ In this secondary analysis, we sought to examine whether these nutrient interventions had an effect on EED biomarker and biochemical measures of systemic inflammation per se, because these are potentially more sensitive indicators of EED and inflammation compared with anthropometric measures and clinical diagnoses of common infections, respectively. Therefore, our specific objectives were to determine whether daily supplementation with zinc and/or multivitamins improves markers of EED, as assessed via anti-flagellin and anti-LPS immunoglobulins, systemic inflammation, as assessed via CRP and AGP, and/or growth, as assessed via IGF-1 and insulin-like growth factor binding protein-3 (IGFBP-3), in a cohort of Tanzanian infants.

## Methods

Infants in this study were participants in a double-blind, placebo-controlled trial (NCT00421668) with a 2 by 2 factorial design (n = 2400) that was conducted between August 2007 and May 2011 in peri-urban Dar es Salaam, Tanzania. The primary objective of the parent trial was to determine whether the daily administration of zinc and/or multivitamins reduced the risk of infectious morbidity compared with a placebo. Detailed methods and results of the parent trial have been published previously,^37^ as have results linking anti-LPS/anti-flagellin immunoglobulins^32^ and CRP/AGP^38^ with poor child growth.

For purposes of randomization into the parent trial, a biostatistician in Boston prepared a list from 1 to 2400 that used blocks of 20 and was stratified by study clinic. Potentially eligible infants born to mothers who were HIV-negative were randomly assigned to 1 of the following 4 study arms at 6 weeks of age: (1) zinc, (2) multivitamins, (3) zinc + multivitamins, or (4) placebo. Infants of multiple births and with congenital anomalies that would interfere with the study procedures were excluded from the parent trial. Caretakers, pharmacy staff, and investigators were blinded to study group assignment. Mothers and infants enrolled in the parent trial were followed for 18 months from randomization or until death or loss to follow-up.

Supplements, manufactured by Nutriset (Malaunay, France), contained an orange-flavored powder and were packaged in a blister pack of 15 each. Details regarding the supplements’ micronutrient make-up have been previously described.^37^ To summarize, zinc capsules contained 5 mg of zinc and multivitamin capsules contained 60 mg of vitamin C, 8 mg of vitamin E, 0.5 mg of thiamine, 0.6 mg of riboflavin, 4 mg of niacin, 0.6 mg of vitamin B6, 130 mg of folate, and 1 mg of vitamin B12. From the time of randomization to 6 months of age, infants received 1 capsule/day, and from 7 months of age to the end of follow-up, 2 capsules were provided daily. As confirmed from field testing, all regimens were identical in taste, smell, and appearance. Compliance was assessed on a monthly basis by study nurses, who counted the number of unconsumed tablets. Median (25th and 75th percentiles) regimen compliance among infants was 96% (91%, 99%) of the allocated regimen.

Blood samples were obtained from children in the parent trial at 6 weeks, 6 months, and 12 months of age. Samples were centrifuged, and serum was removed within 2 hours of blood collection. Aliquots were stored in −80°C freezers until shipped on dry ice for analysis. Of the 2400 infants enrolled in the parent trial, 590 met the following inclusion criteria and were eligible for inclusion in a subanalysis of EED and growth: a blood sample was available at 6 weeks and 6 months of age, and length-for-age z score was ≥ −2 at 6 weeks of age.^32^ For these infants, serum anti-flagellin and anti-LPS immunoglobulins (IgA and IgG) were measured using an enzyme-linked immunosorbent assay as previously reported.^39^ EED biomarker data are reported as optical density–corrected data by subtracting background concentrations determined from the readings in the samples that lacked serum. CRP, AGP, IGF-1, and IGFBP-3 also were measured via enzyme-linked immunosorbent assay (R&D Systems, Minneapolis, Minnesota).

The primary outcomes of this analysis were differences in biomarker concentrations, ie, anti-flagellin and anti-LPS IgA and IgG, CRP, AGP, IGF-1, and IGFBP-3, at 6 months of age between infants who received zinc and/or multivitamins compared with those who did not. A sample size of 590 was calculated for the EED and child growth study to provide 91% power to detect a stunting hazard ratio of 1.5 for every 0.2-unit increase in anti-flagellin IgG and to provide 99% power to detect a stunting hazard ratio of 1.5 for every 0.4-unit increase in anti-LPS IgG.

Data were double entered using Microsoft Access software (Microsoft Corporation, Redmond, Washington) and uploaded to a secured Unix-based server for analyses. Analyses were performed using STATA, version 15 (Stata Corp, College Station, Texas) and SAS, version 9.2 (SAS Institute, Cary, North Carolina) software. Descriptive statistics were calculated to summarize baseline characteristics of the study population. Frequencies and mean ± SDs were reported for categorical and continuous variables respectively. Comparisons of biomarker concentrations between the group that received zinc (ie, received zinc alone as well received zinc + multivitamins) and the group that received multivitamins (ie, received multivitamins alone as well received zinc + multivitamins) were performed using ordinary least squares regression models controlled for baseline values. *P* values < .05 were considered to be statistically significant. Interaction terms were calculated to test for joint effects between zinc and multivitamin factors.

Institutional approval was obtained from the Harvard T.H. Chan School of Public Health Human Subjects Committee; the Muhimbili University of Health and Allied Sciences Senate Research and Publications Committee; the Tanzanian National Institute of Medical Research; and the Tanzanian Food and Drugs Authority. Before enrollment, mothers provided written informed consent to participate in the study. The parent trial was registered at clinicaltrials.gov as NCT00421668.

## Results

The Figure (available at www.jpeds.com) presents the flow diagram for the study. Of the 2400 infants enrolled in the parent trial, 590 infants had EED, systemic inflammation, and growth biomarker data for the 6-week and 6-month time points; 162 infants had EED biomarker data for the 12-month time point. Baseline characteristics by study group for the 590 children in this study are presented in Table I. We observed no significant differences among the 4 groups with regard to maternal, socioeconomic, or child characteristics, with the exception of maternal mid-upper arm circumference (*P* = .01).

**Table I:** Baseline characteristics of 590 Tanzanian infants and their mothers by study group∗

	Placebo (n = 149)	Zinc only (n = 156)	Multivitamins only (n = 142)	Zinc + multivitamins (n = 143)
Maternal characteristics				
Age, y	26.8 ± 5.3	27.2 ± 5.1	26.5 ± 5.1	25.8 ± 5.0
Mid-upper arm circumference, cm	27.1 ± 3.1	27.2 ± 3.4	26.9 ± 3.0	26.1 ± 3.4
Formal education, n (%)				
None	3 (2.0)	6 (3.9)	4 (2.9)	1 (0.7)
1-7 y	108 (73.0)	113 (72.9)	102 (72.9)	106 (74.1)
≥8 y	37 (25.0)	36 (23.2)	34 (24.3)	36 (25.2)
Employment, n (%)				
Housewife without income	94 (64.0)	96 (62.3)	80 (56.7)	76 (53.2)
Housewife with income	49 (33.3)	44 (28.6)	47 (33.3)	53 (37.1)
Other	4 (2.7)	14 (9.1)	14 (9.9)	14 (9.8)
Married or cohabitating with partner, n (%)	135 (91.8)	141 (91.0)	131 (92.9)	130 (92.2)
Previous pregnancies				
None	40 (27.0)	41 (26.5)	43 (30.5)	48 (33.6)
1-4	104 (70.3)	110 (71.0)	93 (66.0)	87 (60.8)
≥5	4 (2.7)	4 (2.6)	5 (3.6)	8 (6.6)
Household possessions,† n (%)				
None	46 (31.1)	44 (28.4)	37 (26.4)	49 (34.3)
1-3	86 (58.1)	83 (53.6)	81 (57.9)	79 (55.2)
>3	16 (10.8)	28 (18.1)	22 (15.7)	15 (10.5)
Infant characteristics				
Age at randomization, wk	5.9 ± 0.29	5.9 ± 0.32	5.9 ± 0.32	5.9 ± 0.36
Male, n (%)	83 (55.7)	73 (46.8)	67 (47.2)	70 (49.0)
Low birth weight (<2500 g), n (%)	4 (2.7)	3 (1.9)	3 (2.1)	2 (1.4)
Born preterm (<37 wk), n (%)	15 (11.4)	16 (11.1)	10 (8.1)	15 (12.0)
Hemoglobin, g/dL	10.5 ± 1.4	10.7 ± 1.3	10.7 ± 1.3	10.5 ± 1.3
Length-for-age z score at 6 wk of age	−0.16 ± 0.92	−0.18 ± 1.02	−0.17 ± 1.10	−0.07 ± 1.00
Weight-for-length z score at 6 wk of age	0.02 ± 1.18	0.01 ± 1.26	0.02 ± 1.21	−0.11 ± 1.26
Weight-for-age z score at 6 wk of age	−0.16 ± 0.82	−0.18 ± 0.85	−0.18 ± 0.91	−0.17 ± 0.90

∗ Values are means ± SDs or percentages. No significant differences were observed in baseline characteristics among treatment groups (*P* > .05) with the exception of maternal mid-upper arm circumference (*P* = .01).

† From a list that included a sofa, television, radio, refrigerator, and fan.

Table II shows serum concentrations of EED, systemic inflammation, and growth biomarkers at 6 weeks and 6 months of age, and at 12 months of age for EED biomarkers. Mean EED biomarker concentrations increased over the first year of life (*P*-trend <.001). However, with the exception of IGFBP-3 concentrations at 6 months of age (which were significantly lower in children who received zinc compared with those who did not [981.13 ± 297.59 vs 1019.10 ± 333.01, *P* = .03]) we did not observe any significant differences in biomarker concentrations of EED, systemic inflammation, or growth in infants who received zinc compared with infants who did not receive zinc at any of the assessed time points (*P* ≥ .05 for all). Furthermore, with the exception of anti-LPS IgG concentrations at 6 months of age, which were significantly greater in infants who received multivitamins compared with those who did not (1.41 ± 0.61 vs 1.26 ± 0.65, *P* = .006), we did not observe any significant differences in EED, systemic inflammation, or growth biomarkers in infants who received multivitamins compared with infants who did not receive multivitamins at any of the assessed time points (*P* ≥ .05 for all).

**Table II:** Effect of daily zinc and multivitamins on biomarkers of EED, systemic inflammation, and growth in Tanzanian infants

	Received zinc∗		*P*§	Received multivitamins†		*P*§	*P*_*int*_¶
	Yes‡	No		Yes	No		
6-wk values (n = 590)							
Flagellin IgA∗∗	0.28 ± 0.27	0.32 ± 0.28	.05	0.30 ± 0.30	0.30 ± 0.26	.85	.81
Flagellin IgG	0.51 ± 0.35	0.50 ± 0.31	.79	0.51 ± 0.32	0.50 ± 0.34	.76	.57
LPS IgA	0.41 ± 0.43	0.45 ± 0.44	.24	0.44 ± 0.44	0.42 ± 0.43	.60	.81
LPS IgG	0.80 ± 0.56	0.77 ± 0.46	.44	0.80 ± 0.77	0.77 ± 0.50	.39	.39
AGP, g/L	37.91 ± 21.55	39.41 ± 21.85	.41	38.96 ± 21.95	38.37 ± 21.49	.75	.94
CRP, mg/L	1.01 ± 2.87	1.34 ± 4.57	.35	1.13 ± 3.36	1.23 ± 4.26	.79	.69
IGF-1, ng/mL	40.88 ± 11.83	38.98 ± 11.88	.05	39.51 ± 12.20	40.32 ± 11.57	.41	.72
IGFBP-3, ng/mL	989.30 ± 330.87	963.76 ± 324.66	.35	973.17 ± 342.75	979.62 ± 312.97	.81	.49
6-mo values (n = 590)							
Flagellin IgA	0.61 ± 0.41	0.62 ± 0.39	.83	0.61 ± 0.38	0.62 ± 0.43	.79	.27
Flagellin IgG	0.86 ± 0.49	0.85 ± 0.47	.82	0.89 ± 0.47	0.83 ± 0.49	.11	.90
LPS IgA	0.96 ± 0.63	0.94 ± 0.60	.74	0.98 ± 0.60	0.92 ± 0.63	.32	.95
LPS IgG	1.33 ± 0.63	1.34 ± 0.63	.77	1.41 ± 0.61	1.26 ± 0.65	.006	.12
AGP, g/L	72.58 ± 34.88	74.51 ± 36.01	.47	74.35 ± 36.84	72.81 ± 34.12	.54	.99
CRP, mg/L	3.43 ± 8.17	3.48 ± 7.88	.95	3.58 ± 8.78	3.33 ± 7.23	.73	.26
IGF-1, ng/mL	24.95 ± 10.03	24.62 ± 10.07	.98	24.79 ± 10.21	24.77 ± 9.90	.79	.51
IGFBP-3, ng/mL	981.13 ± 297.59	1019.10 ± 333.01	.03	1001.07 ± 318.28	999.70 ± 314.99	.80	.93
12-mo values (n = 162)							
Flagellin IgA	1.19 ± 0.59	1.25 ± 0.65	.66	1.18 ± 0.61	1.27 ± 0.62	.39	.52
Flagellin IgG	1.55 ± 0.45	1.59 ± 0.45	.53	1.54 ± 0.41	1.61 ± 0.49	.33	.96
LPS IgA	1.28 ± 0.64	1.34 ± 0.68	.60	1.28 ± 0.65	1.35 ± 0.67	.54	.51
LPS IgG	1.58 ± 0.45	1.56 ± 0.44	.78	1.57 ± 0.46	1.57 ± 0.42	.96	.46

∗ Received zinc “yes” denotes children who received zinc alone as well as those who received zinc and multivitamins. Received zinc “no” denotes all children who received multivitamins alone and those who received the placebo.

† Received multivitamins “yes” denotes children who received multivitamins alone as well those who received zinc and multivitamins. Received multivitamins “no” denotes all children who received zinc alone and those who received the placebo.

‡ Values are mean ± SD.

§ *P* values at 6 months and 12 months obtained from ordinary least squares regression models controlled for baseline concentrations (ie, at 6 weeks).

¶ *P*-interaction term.

∗∗ Units are optical density.

Finally, we observed no statistically significant differences in changes in anti-flagellin, anti-LPS, AGP, CRP, or IGF-1 concentrations from 6 weeks to 6 months or in changes in anti-flagellin and anti-LPS from 6 to 12 months according to receipt of either zinc or multivitamins (*P* ≥ .05 for all) (Table III; available at www.jpeds.com). Changes in IGFBP-3 concentrations from 6 weeks to 6 months were significantly lower in children who received zinc compared with those who did not (−9.17 ± 323.07 vs 54.49 ± 330.41, *P* = .02). No difference was observed in changes in IGFBP-3 concentrations from 6 weeks to 6 months in children who received multivitamins compared with those who did not.

## Discussion

In this study of Tanzanian infants, we found that neither EED, as assessed via anti-flagellin and anti-LPS immunoglobulins, nor systemic inflammation, as assessed via CRP and AGP, was ameliorated by supplementation with zinc and/or multivitamins. Furthermore, supplementation with zinc and/or multivitamins had generally no effect on biomarkers of growth, as assessed via IGF-1 and IGFBP-3.

We did find that anti-LPS IgG concentrations at 6 months of age were significantly greater in infants who received multivitamins compared with those who did not. This finding could potentially be related to a more robust immune response in the setting of vitamin supplementation, especially among populations with marginal vitamin status.^29^ However, this single finding was not noted at 6 weeks or 12 months of age, which raises the possibility that it was a chance finding. We also found that IGFBP-3 concentrations at 6 months of age were significantly lower in children who received zinc compared with those who did not, but we unfortunately did not have IGFBP-3 data at 12 months of age. This is also potentially a chance finding, as we are aware of no other studies which have demonstrated that zinc supplementation has a negative effect on either growth hormone secretion or linear growth.

Our results are consistent with the results from our parent trial study, which found no significant effect of zinc and/or multivitamin supplementation on growth outcomes (ie, underweight, stunting, or wasting)^36^ but does add to the growing body of literature on trials to prevent or ameliorate EED. To date, trials involving probiotics,^40^ antibiotics,^41^ alanyl-glutamine,^42^ long-chain polyunsaturated fatty acids,^43^ vitamin A supplementation,^44^ and albendazole,^27^ have been conducted, all demonstrating only limited to moderate improvements in small intestine function.

It is worth noting that in many of the aforementioned trials, EED was assessed via the L:M test, which remains the most commonly used test for EED, whereas we assessed EED using serum anti-flagellin and anti-LPS immunoglobulins. The bacterial protein flagellin, which mediates bacteria motility, and LPS, a major structural component of bacteria, are typically excluded from absorption by the epithelium except in the case of intestinal barrier dysfunction. The translocation of LPS and flagellin across the intestinal mucosa activates the adaptive immune response resulting in the presence anti-flagellin and anti-LPS antibodies.^45^, ^46^ Previously, elevations in anti-flagellin and anti-LPS Ig concentrations have been observed in other types of chronic enteric inflammatory conditions, including short bowel syndrome, Crohn's diseases, and others.^39^, ^47^

To date, numerous other EED biomarkers have been proposed, which measure different domains of EED, eg, intestinal permeability, absorption, or inflammation.^48^ In addition, some biomarkers, including the antibody concentrations we studied, have a relatively longer half-life than other, more acute markers (eg, serum protein concentrations, stool inflammatory markers), so the relationship between the time of EED exposure and biomarker outcome may vary substantially. A study of 539 Bangladeshi children by Campbell et al demonstrated low correlations among a panel of proposed EED biomarkers,^49^ a finding that illustrates the difficulties of defining EED with noninvasive biomarkers. Due to limitations of blood volume and other sample availability, our study was limited by its reliance on only one class of EED biomarker, which might explain our inability to detect significant effects of the intervention.

To our knowledge, this is one of only a few studies to examine the potential of zinc and/or multivitamin supplementation to ameliorate EED and/or systemic inflammation, especially in a resource-limited setting. Notably, our results contrast with findings from a limited number of studies that have shown an affect, albeit modest, of zinc on ameliorating EED. In a randomized, double-blind, placebo-controlled trial conducted in rural Malawi, asymptomatic, 1- to 3-year-old children received either a single dose of albendazole, a 14-day course of 20 mg zinc sulfate, or a placebo. At 34 days after completion of the assigned regimen, the L:M had increased more in the placebo group (0.12 ± 0.31) than in the zinc group (0.03 ± 0.20; *P* < .03) or the albendazole group (0.04 ± 0.22; *P* < .04).^27^ In addition, in a study by Bates et al conducted in rural Gambia, children 0.57-2.30 years old received either 70 mg of zinc or placebo twice weekly for 1.25 years.^50^ The lactulose:creatinine level was improved (*P* < .02) with zinc supplementation, but the L:M level was not improved.

However, our results are consistent with other studies that have shown no significant effect of zinc or multivitamin supplementation in ameliorating EED in young children.^51^ In a randomized, double-blind, placebo-controlled trial conducted in rural Malawi, asymptomatic, 1- to 3-year-old children in the intervention group received a single dose of albendazole, 14 days of zinc supplementation at enrollment and after 20 weeks, and a daily micronutrient powder throughout the 24 weeks of study. Increases in the L:M did not differ between the intervention group and the placebo group after 12 weeks (0.071 units vs 0.073 units, *P* = .87) or after 24 weeks (0.088 units vs and 0.080 units, *P* = .19).^52^

Several strengths and limitations of the study are worthy of mention. This was a rigorous double-blind, placebo-controlled trial in a population at high risk of EED. However, in addition to measuring only a single class of EED biomarkers, limitations of this study include a relatively small sample size, particularly at the 12-month time-point (n = 162), and the absence of biomarker concentrations beyond either the 6- or 12-month time-point in all children, after which there could potentially be greater benefits from zinc.

In conclusion, daily supplementation with zinc and/or multivitamins beginning at 6 weeks of age failed to ameliorate EED and/or systemic inflammation during infancy in a sample of Tanzanian infants. Therefore, although zinc supplementation may indeed result in considerable reductions in the incidence of diarrhea and modest improvements in growth in young children in LMICs, our results suggest that the mechanism is not readily demonstrable with 6-week, 6-month, and 12-month serum sample testing for these biomarkers. Based on our findings, we recommend that other strategies for ameliorating EED and systemic inflammation, besides preventive zinc and multivitamin supplementation, be prioritized for future trials. We also recommend that future studies employ a range of EED biomarkers to better assess the ability of proposed interventions to ameliorate different domains of EED.

## Figures and Tables

**Figure:**
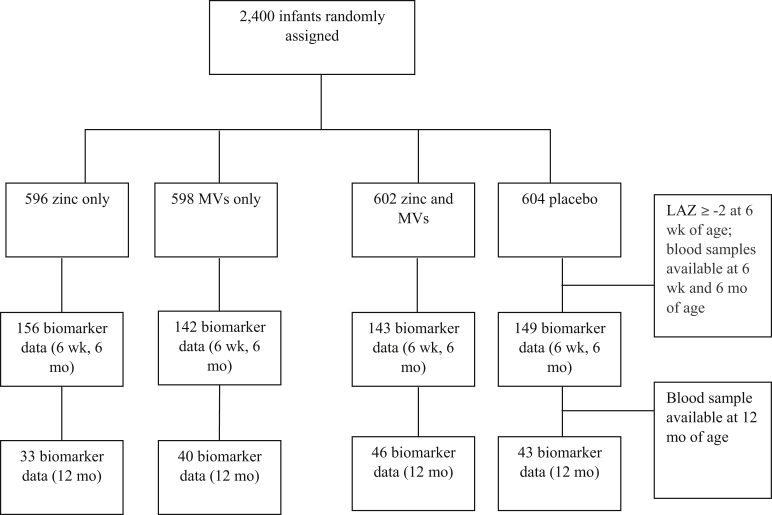
Flowchart of study enrollment and follow-up evaluation. *LAZ*, length-for-age z score.

**Table III:** Effect of daily zinc and multivitamins on changes in biomarkers of EED, systemic inflammation, and growth in Tanzanian infants

	Received zinc∗		*P*§	Received multivitamins†		*P*§	*P*_*int*_¶
	Yes‡	No		Yes	No		
Change from 6 wk to 6 mo (n = 590)							
Flagellin IgA∗∗	0.33 ± 0.48	0.30 ± 0.49	.37	0.31 ± 0.49	0.32 ± 0.49	.74	.29
Flagellin IgG	0.35 ± 0.60	0.35 ± 0.54	.96	0.38 ± 0.55	0.33 ± 0.58	.23	.66
LPS IgA	0.54 ± 0.73	0.49 ± 0.73	.36	0.53 ± 0.74	0.50 ± 0.72	.58	.93
LPS IgG	0.57 ± 0.76	0.53 ± 0.78	.49	0.61 ± 0.76	0.50 ± 0.77	.08	.49
AGP, g/L	34.43 ± 37.02	35.64 ± 36.29	.70	35.65 ± 37.53	34.46 ± 35.79	.70	.82
CRP, mg/L	2.72 ± 9.21	2.70 ± 9.43	.98	2.89 ± 10.06	2.54 ± 8.54	.71	.28
IGF-1, ng/mL	−15.60 ± 13.44	−14.39 ± 13.66	.28	−14.61 ± 13.26	−15.35 ± 13.85	.51	.87
IGFBP-3, ng/mL	−9.17 ± 323.07	54.49 ± 330.41	.02	28.58 ± 352.95	17.89 ± 302.55	.69	.55
Change from 6 mo to 12 mo (n = 162)							
Flagellin IgA	0.56 ± 0.66	0.60 ± 0.66	.73	0.57 ± 0.64	0.59 ± 0.68	.80	.86
Flagellin IgG	0.69 ± 0.56	0.72 ± 0.60	.72	0.66 ± 0.57	0.76 ± 0.59	.29	.85
LPS IgA	0.34 ± 0.73	0.32 ± 0.91	.87	0.26 ± 0.84	0.40 ± 0.81	.26	.77
LPS IgG	0.19 ± 0.66	0.16 ± 0.75	.81	0.09 ± 0.71	0.27 ± 0.69	.12	.39

∗ Received zinc “yes” denotes children who received zinc alone as well as those who received zinc and multivitamins. Received zinc “no” denotes all children who received multivitamins alone and those who received the placebo.

† Received multivitamins “yes” denotes children who received multivitamins alone as well those who received zinc and multivitamins. Received multivitamins “no” denotes all children who received zinc alone and those who received the placebo.

‡ Values are mean ± SD.

§ *P* values obtained from 2-sided *t* test.

¶ *P*-interaction term.

∗∗ Units are optical density.

## Supplementary Data

CONSORT Checklist
